# Effect of physical stimuli on hair follicle deposition of clobetasol-loaded Lipid Nanocarriers

**DOI:** 10.1038/s41598-019-56760-w

**Published:** 2020-01-13

**Authors:** Tamara Angelo, Nesma El-Sayed, Marijas Jurisic, Aljoscha Koenneke, Guilherme M. Gelfuso, Marcilio Cunha-Filho, Stephania F. Taveira, Robert Lemor, Marc Schneider, Tais Gratieri

**Affiliations:** 1 Laboratory of Food, Drugs, and Cosmetics (LTMAC), University of Brasilia, Brasília, Brazil; 2 Biopharmaceutics and Pharmaceutical Technology, Saarland University, Saarbrücken, Germany; 3 Department of Pharmaceutics, Faculty of Pharmacy, Alexandria University, Alexandria, Egypt; 4 Laboratory of Nanosystems and Drug Delivery Devices (NanoSYS), School of Pharmacy, Universidade Federal de Goiás (UFG), Goiás, Brazil; 5 Saarland University of Applied Sciences, Saarbrücken, Germany

**Keywords:** Drug development, Translational research

## Abstract

Clobetasol propionate (CLO) is a potent glucocorticoid used to treat inflammation-based skin, scalp, and hair disorders. In such conditions, hair follicles (HF) are not only the target site but can also act as drug reservoirs when certain formulations are topically applied. Recently, we have demonstrated nanostructured lipid carriers (NLC) containing CLO presenting epidermal-targeting potential. Here, the focus was evaluating the HF uptake provided by such nanoparticles in comparison to a commercial cream and investigating the influence of different physical stimuli [*i.e*., infrared (IR) irradiation (with and without metallic nanoparticles-MNP), ultrasound (US) (with and without vibration) and mechanical massage] on their follicular targeting potential. Nanosystems presented sizes around 180 nm (PdI < 0.2) and negative zeta potential. The formulation did not alter skin water loss measurements and was stable for at least 30 days at 5 °C. Nanoparticles released the drug in a sustained fashion for more than 3 days and increased passively about 40 times CLO follicular uptake compared to the commercial cream. Confocal images confirmed the enhanced follicular delivery. On the one hand, NLC application followed by IR for heat generation showed no benefit in terms of HF targeting even at higher temperatures generated by metallic nanoparticle heating. On the other hand, upon US treatment, CLO retention was significantly increased in deeper skin layers. The addition of mechanical vibration to the US treatment led to higher follicular accumulation compared to passive exposure to NLC without stimuli. However, from all evaluated stimuli, manual massage presented the highest follicular targeting potential, driving more than double the amount of CLO into the HF than NLC passive application. In conclusion, NLC showed great potential for delivering CLO to HF, and a simple massage was capable of doubling follicular retention.

## Introduction

Skin, hair, and scalp-related dermatological inflammatory pathologies have been treated for decades with oral and topical glucocorticoids. Among the topical drugs under study, clobetasol propionate (CLO) stands out as the most potent one^1^. Due to its vasoconstricting, anti-inflammatory, immunosuppressive and antiproliferative effects, the drug is useful in the treatment of conditions such as eczema, atopic dermatitis, alopecia areata, frontal fibrosing alopecia, psoriasis and lichen planopilaris^2–4^. Nevertheless, continuous use of typical CLO dermatological formulations may present local side effects as skin atrophy, pruritus, folliculitis, and telangiectasia^5–7^. Even though topical treatment results in fewer adverse effects when compared to oral or parenteral administration, formulations capable of controlling drug release while targeting and enhancing drug penetration to specific skin layers can provide additional therapeutic benefits.

Previous studies from our group have shown CLO-loaded nanostructured lipid carriers (NLC), produced with 1/5^th^ of the drug dose used in commercial formulations, presented higher skin permeation with an epidermal targeting potential^8^, which could prevent some of the above-mentioned adverse effects in a clinical application. Still, for inflammation-caused scalp diseases, targeted delivery to hair follicles (HF) would definitely be an even more promising strategy to minimize local adverse effects. HF could act as an entry and storage site, promoting controlled and prolonged release of the drug^9^, which could also be advantageous in improving patient compliance. In fact, this has been suggested in recent studies evaluating follicle targeting potential of different nanocarriers containing CLO^10^. In such a study, from the three types of polymeric nanocarriers studied (i.e., nanospheres, nanocapsules, lipid-core nanocapsules), follicular uptake was higher for the nanocapsules, which contained a liquid lipid interior. Such enhanced uptake of the most flexible nanoparticles may suggest even better results for NLC. Besides, the mentioned study confirmed previous positive effects of mechanical massage upon follicular penetration^11^. The explanation of such effect was the process of moving the hair by massaging the skin acts like a ‘gear pump,’ transporting the nanoparticles into the HF, *i.e*., into the hair shaft, by mimicking the natural movement of the hair together with the scaly structure of the hair cuticula^12^. Therefore, according to such a theory, the mechanical movement of the hair drives the particles. An intriguing unanswered question is whether inflicting any kind of “perturbance” to the formulation rather than the hair shafts would elicit nanoparticle movement and also enhance follicular penetration by some “active input.” Indeed, we have previously observed that even a slight mechanical movement triggered by an effervescent reaction could lead to enhanced follicular deposition of minoxidil^13^. However, because of the lipophilic nature of CLO (Log P = 3.0), NLC comprises a much more attractive drug delivery system than solid formulations^14,15^.

Thus, the strategy could be to apply an external physical stimulus to increase the thermodynamic energy of the system. One of the more ancient ways to increase systems thermodynamic energy would be to heat the formulation using some radiation-emitting device. In fact, infrared irradiation (IR) studies suggest higher hair growth in patients with alopecia areata^16^, meaning that it could be a safe approach. Still, the temperature should be tightly controlled, so that the generated heat reaches the HF without causing damage.

Another external energy source that could be capable of moving nanoparticles through the medium in which it propagates is ultrasound (US). The use of US in cutaneous therapies, known as sonophoresis or phonophoresis, has also demonstrated to be safe^17^. The main mechanisms involved in increasing permeation of substances through the skin and skin appendages by the use of US is cavitation, which occurs when gas bubbles are formed in unfilled spaces or preexisting bubbles move, contract, expand or contort due to US acoustic pressure oscillations, causing stratum corneum (SC) disorganization or moving content into the appendices^18^.

Thus, we hypothesized (i) NLC would be a promising formulation for targeted follicular delivery of CLO and (ii) an increase in nanoparticle movement elicited by an external physical stimulus could further increase follicle uptake. To test these hypotheses, first, the follicular target potential of NLC was investigated in both *in vitro* and *ex vivo* permeation experiments. Then, *in vitro* permeation experiments in which formulations were submitted to laser irradiation (with and without magnetic nanoparticles) and US (with and without device vibration) were performed. Magnetic nanoparticles were added to some of the IR experiments to achieve a controlled and slightly higher IR absorption and thus a stronger hyperthermal effect. Results were then compared to either passive delivery or formulation application followed by a manual massage stimulus.

## Material and Methods

### Material

CLO, sodium taurodeoxycholate, sodium dodecyl sulfate (SDS), dialysis membrane 12,000 Da MWCO, 2-morpholino-ethanesulfonic acid monohydrate (MES) and 4-(2-hydroxyethyl)-1piperazineethanesulfonic acid (HEPES) were all purchased from Sigma-Aldrich (Steinheim, Germany). Stearic acid and oleic acid used in NLC production were purchased from Merck (Darmstadt, Germany). Soybean lecithin S100 was obtained from Lipoid (Ludwigshafen, Germany). A generic drug in the form of a cream containing 0.05% of CLO (commercial cream) (EMS laboratory, SP, Brazil, batch 468593) was purchased locally. It is comprised of non-ionic self-emulsifying wax, decyl oleate, cetostearyl alcohol ethoxylate, propylene glycol, glycerol, phenoxyethanol, paraben, and purified water. For differential tape stripping process Scotch Book Tape No. 845 (3 M, St. Paul, MN, USA) and Super Bonder cyanoacrylate adhesive, Loctite-Henkel (Dublin, Ireland) were used. Rhodamine 6 G (R6G) was purchased from Fluka (Steinheim, Germany). Fetal bovine serum (BioWhittaker, FBS) was supplied by Lonza (Basel, Switzerland). Dulbecco’s modified Eagle’s medium (DMEM) and penicillin-streptomycin were obtained from Fisher Scientific (Hampton, NH, USA). Solvents with chromatographic grade were supplied by Tedia Brazil (Rio de Janeiro, Brazil) or VWR Chemicals (Radnor, USA). Ultrapurified water (Millipore, Illkirch-Graffenstaden, France) was used for all analyses. Freshly excised porcine ears were gently donated by a local slaughterhouse (Bonasa, Brasilia, Brazil or Färber, Zweibrücken, Germany).

### Preparation of NLC

CLO-loaded NLC (NLC-CLO) were obtained, as previously described^19^. Briefly, 300 mg stearic acid, 100 mg oleic acid, 200 mg lecithin, and 50 mg sodium taurodeoxycholate were heated to 80 °C, and CLO was added to the molten material. To obtain a hot microemulsion, 250 μL water was added. The microemulsion was then dripped in a MES buffer solution (0.1 mol/L, pH 5.5) under vigorous stirring (13,500 rpm for 10 min) using an Ultra-turrax^®^ T-25 homogenizer with S25N-25F dispersant (IKA, Staufen, Germany). The final pH of the formulation was 5. Microemulsion/buffer ratio was 1:20 (v/v). The dispersion had a total lipid content of 2% (w/v), stabilized by 1.25% (w/v) of surfactant (lecithin: taurodeoxycholate, 4:1 ratio). The stearic acid: oleic acid ratio in the NLC was 3:1. The theoretical drug concentration was 0.05%.

In order to obtain fluorescent NLC, the preparation was as before replacing CLO by R6G.

### NLC characterization

NLC-CLO morphology was evaluated by scanning electron microscopy (SEM). For that, the formulation was diluted 1:500 (v/v) in ultrapure water. A drop of the suspension was deposited on a silica wafer, which was mounted on a metallic support with the aid of carbon tape. The support was placed in a desiccator overnight for liquid evaporation. The samples were then coated with gold (~10 nm) under an argon atmosphere using the Q150RES spray coater (Quorum Technologies Ltd., Laughton, UK). SEM images were obtained by an EVO HD15 microscope (Carl Zeiss Microimaging, Jena, Germany). In order to assess size distribution, polydispersity index (PdI) and zeta potential, 10 μL of each formulation was suspended in 990 μL of ultrapure water, vortexed for 30 seconds and taken to the apparatus for analysis (Zetasizer Nano ZS, Malvern, Worcestershire, United Kingdom).

Differential scanning calorimetry (DSC) was performed in aluminum pans under nitrogen atmosphere (flow rate of 50 mL min^−1^) at a heating rate of 10 °C min^−1^ from 30 to 300 °C using a DSC-60 (Shimadzu, Kyoto, Japan). Tests were performed on individual compound and NLC-CLO.

Total drug amount, entrapment efficiency (EE) and drug loading (DL) were determined according to Eqs. 1 and 2 using the analytical procedure described below. The formulations were stored in closed, clear glass vials under 5, 25 and 40 °C, and their stability was evaluated for 0, 7, 15 and 30 days (n = 3).

1 $$Entrapment\,efficiency(E{E}_{( \% ,w/w)})=\frac{Total\,drug\,amount\,-\,free\,drug}{Total\,drug\,amount}\times 100$$

2 $$Drug\,Loading(D{L}_{( \% ,w/w)})=\frac{Total\,drug\,amount-free\,drug}{Total\,amount\,of\,lipids}\times 100$$

### Drug release study

*In vitro* release assays were performed using Franz-type diffusion cells (FD-C). A dialysis membrane was assembled between the donor (15 mm diameter) and the acceptor compartments (volume capacity of 12 mL). The acceptor compartment was filled with 0.5% (w/v) SDS aqueous solution to ensure sink conditions^19^. At the donor compartment, it was placed (i) NLC-CLO (500 μL), (ii) free drug solution (ethanol-water 45:55%, v/v) (500 μL) or (iii) the commercial cream (500 mg) (n = 6 for each formulation). Cells were incubated at 32 °C and maintained under magnetic stirring at 300 rpm. Samples of the acceptor solution were collected at 1, 2, 4, 6, 12, 24, 48, 72, 96 and 120 h and analyzed by the HPLC method described below.

### Cell viability

For cell viability studies, HaCat human keratinocytes^20^ were cultured in DMEM cell culture flasks containing 10% fetal bovine serum and 100 U/mL penicillin-streptomycin. The flasks were placed in an incubator at 37 °C and 5% CO_2_ environment. The culture medium was changed every 48 h. After verification of cell growth, the medium was removed and cells were washed with 10 mL of D-PBS. Then 3.5 mL of trypsin was added and the flask returned to the incubator for 6–8 minutes. When the cells were detached from the bottom of the flask, 7 mL of the medium was added and the contents were transferred to a 15 mL falcon tube, which was centrifuged at 300 rcf for 4 minutes. After removal of the supernatant, cells were resuspended with 5 mL of medium and cell concentration was determined using a Neubauer counting chamber. From this medium, 12 mL of cell suspension was prepared in a new falcon to provide 96-well plate plating at a concentration of 10^4^ cells/well with a volume of 200 µL/well. After plating, cells were incubated for 3 days. At the end of the period, the culture medium was removed and the cells adhered to the bottom of the plate were washed twice with pH 7.4 phosphate buffer using 200 µL each time.

Then, for the cell exposure to the formulations, 200 µL of each sample was added into separate wells: phosphate buffer pH 7.4 (negative control), Triton X-100 2% (positive control), or samples of the formulations: (i) 0.05% CLO solution in ethanol: water (45:55), and (ii) NLC-CLO. Each sample was added in triplicate. After 4 h of incubation the samples were removed, the wells were washed with 200 µl phosphate buffer pH 7.4 and MTT reagent (5 mg/mL) in phosphate buffer was added at 1:10 (v/v) ratio. The plate was incubated again. After additional 4 h, the reagent was removed and 100 µL of DMSO was added to each well of the plate for solubilization of formazan crystals produced by metabolic active cells. The plate was protected from light with aluminium foil, incubated for 20 minutes and had its absorbance measured at a wavelength of 550 nm on a Tecan Infinite M200 plate reader (Tecan Deutschland GmbH, Crailsheim, Germany). The complete process was performed 3 times, totaling 9 replicates for each sample.

### Fluorescent NLC permeation study and confocal microscopy

NLC-R6G or its control (R6G in 45% (v/v) EtOH_(aq)_) were subjected to the *in vitro* permeation test using FD-C. However, neither differential tape stripping nor quantification was performed. After 12 h of permeation, each formulation was removed and the skin was cleaned with cotton and then frozen using CO_2_. The skin samples were then stored at −80 °C for further manual scalpel cutting. Each skin replicate (n = 3) generated about ten sections, which were placed on glass slides and analyzed immediately by confocal microscopy (LSM 710 Zeiss, Jena, Germany)^21^. To obtain the images, EC-Neofluar 10×/0.30 M27 objective lens and MBS 458/561 beam splitter were used. The excitation wavelength was 561 nm, and the emission detection range was 600–700 nm.

Permeation of NLC-R6G was observed in *ex vivo* porcine skin samples for 7 days. Porcine ear skin samples were collected at a slaughterhouse; however, they were not frozen and were used immediately after collection.

The methodology of human HF organ culture (HFOC), and the method *of human organotypic skin explant in culture* (HOSEC) were adapted for this study^22,23^. As so, full-thickness porcine skin was mounted in FD-C, and the acceptor compartment was filled with a culture medium consisting of DMEM, FBS, and penicillin-streptomycin (100:10:1). The collecting channel of the cell was closed with cotton.

NLC-R6G was deposited in the donor compartment, and the cell was placed in an incubator at 37 °C and 5% CO_2_ environment. After 12 h, the formulation was withdrawn, and the skin surface was cleaned with cotton. The culture medium was changed every 48 h. Skin samples were collected at 1, 3, 6, 12, 24, 72 and 168 h. Skin preparation for confocal microscopy visualization was performed as previously described.

### *In vitro* cutaneous permeation

FD-Cs were placed in an incubator at 32 °C and only after ambiance for at least 30 min the experiments started. Transepidermal water loss (TEWL) measurements were also performed after *in vitro* skin permeation experiments to verify if the formulations promoted any significant change in water loss, indicating changes in skin barrier function.

### Passive permeation

For passive permeation studies, in order to study infinite dose effect, NLC-CLO formulation (500 μL), free drug solution (ethanol-water 45:55%, v/v) (500 μL) or commercial cream formulation (500 mg) was placed in the donor compartment (n = 6 for each formulation), all containing theoreticall 250 µg CLO. FD-Cs were then left in an incubator at 32 °C, for 12 h.

### IR irradiation

Magnetite nanoparticles (MNP) coated with oleic acid were developed for later incorporation into the NLC-CLO formulation for laser irradiation studies. Initially, nanoparticles of magnetite were synthesized using the protocol proposed by Sun *et al*.^24^. For that, 2 mmol of tris(acetylacetonate)iron (III) and 10 mmol of 1,2-hexadecanediol were mixed with 6 mmol of oleic acid, 6 mmol of oleylamine and 10 mL of benzyl ether. The mixture was stirred, heated to 100 °C and degassed under vacuum in an anhydrous environment. After the mixture was exposed to the nitrogen atmosphere, the temperature was raised at a rate of 3.3 °C/min until 200 °C, and this temperature was maintained for 2 h under reflux. Thereafter, the temperature was again increased at the same rate until it reached 300 °C and held for a further 1 h. The solution was then cooled to room temperature, ending the synthesis. After the addition of 25 mL of ethanol, the mixture (500 mL) was centrifuged. The supernatant was discarded, and the precipitate was dispersed in toluene with 50 μL of oleic acid and 50 μL of oleylamine. The mixture was re-precipitated with ethanol and finally redispersed in 20 mL of hexane, resulting in MNP dispersed in the organic solvent.

In order to obtain nanoparticles dispersible in water, a new layer of oleic acid was added. Therefore, 1% oleic acid was added to ultrapure water at pH 10.0 adjusted with NaOH. MNP suspension in organic solvent was added to this solution (1:1, v/v). The vial was taken to Vibra-Cell VC 750 sonicator (Sonics, Newtown, USA) with amplitude of 10%. The opened flask was then heated to 80 °C with stirring for 4 h in Thermomixer (Eppendorf, Hamburg, Germany) arranged in a hood for evaporation of the organic solvent. After cooling, the mixture was washed with ultrapure water, using VivaSpin (10,000 rcf), to remove excess oleic acid. At the end of the process, MNP was obtained in aqueous dispersion. To determine the MNP absorption wavelength range, the formulation was diluted 10 and 200 times in water and scanned on a Lambda 35 UV/VIS spectrophotometer (PerkinElmer, Waltham, USA). For comparative purposes, a skin extract was also analyzed. MNP morphology was observed by transmission electron microscopy (TEM). A drop of the suspension was deposited on a copper grid positioned on a slide. After excess liquid was carefully removed with paper, the sample was allowed to dry. A JEM 2011 microscope (JEOL, Freising, Germany) and MultiScan Camera Model 794 camera (Gatan, Pleasanton, CA, United States of America) were used. To obtain the images, 200 keV were used.

In order to perform experiments under hyperthermal conditions, MNP were dispersed in the previously produced NLC formulation, in the final concentration of 1 mg of MNP for each 1 mL of NLC-CLO formulation.

Medilas D SkinPulse S diode laser (Dornier MedTech, Munich, Germany) with an emission at 940 nm was used for irradiation. It had its optical fiber positioned 4 cm from the skin since this was the necessary distance for the pilot light to cover the entire area of exposed skin (1.77 cm^2^) in the FD-C. LabMax-TOP Laser Power and Energy Meter device (Coherent, Santa Clara, CA, USA) were used to measure power attenuation at the given distance.

Heating effect produced by the laser was evaluated by adding 500 μL of NLC-CLO or NLC-CLO + MNP (1 mg/mL) in FD-C donor compartment and irradiating the sample at different intensities for 5 min in continuous mode. The temperature rise of the system was measured with an optical Fiber Optic Temperature Sensor (Optocon, Dresden, Germany) and the data were collected by FoTemp software FCT-0139 (Optocon, Dresden, Germany). Three different FD-C with skin samples were used for each measurement.

After determination of the ideal conditions of potency and time, hyperthermia experiments in combination with skin permeation studies were conducted. Laser irradiation was performed (i) at the beginning, immediately after application of the formulation or (ii) at the end of the protocol, after 12 h at 32 °C (n = 6).

### Permeation effect due to ultrasound application

To evaluate the effect of US on nanoparticles follicular targeting, a dermatological home device FC 80 (Beurer, Ulm, Germany) was used, with a frequency of 5 MHz and an intensity of 1.2 W/cm^2^. The device operates in pulsed mode and has been modified so that the extra mechanical vibration produced by the system could be deactivated. Thus, the equipment could be used in two distinct functions: (i) only pulsed US; or (ii) pulsed US with mechanical vibration.

The skin was laid on filter paper soaked in HEPES buffer pH 7.4 and positioned on a glass slide. The exposed skin area was delimited by laminated adhesive tape, with a circle of 15 mm in diameter. After administration of 40 μL of NLC-CLO on the skin, US with or without vibration was applied for 3 min. The skin was then placed in a FD-C, the formulation volume was completed to 500 μL, and the cell left in an incubator at 32 °C for 12 h.

### Permeation effect due to application of massage

Skin was delimited, as described in the previous section. Then, 40 μL of NLC-CLO formulation was applied and massaged clockwise for 3 min, using the same index finger of the same person. At the end of the massage, the skin was mounted in the FD-C, the volume of the formulation was completed to 500 μL, and *in vitro* skin permeation studies were conducted as described before.

For all the cutaneous permeation experiments, at the end of 12 h at 32 °C, the acceptor solution was collected, the skin was removed from the FD-C, excess formulation was removed, and the skin was cleaned with cotton for further analyses.

### Analytical procedure

In order to evaluate the amount of penetrated CLO after cutaneous permeation studies, differential tape stripping method^25^ was performed (Supplement 1). Tapes with SC, tapes with HF or remaining skin (RS) were placed in a vial with 5 mL methanol for 3 h under magnetic stirring (300 rpm). The filtered skin extracts were then quantified.

CLO quantification was performed using a validated HPLC-UV method^26^. Briefly, a RP-C18 column (4.6 mm × 15 cm, 5 μm) was used, with mobile phase constituted of methanol–acetonitrile–water (50:15:35 v/v), a flow rate of 1.2 mL/min, oven temperature of 30 °C, injection volume of 50 μL and detection at 240 nm.

### Data analysis

All data are expressed as mean ± standard deviation. Statistical significance of the data was evaluated either by analysis of the variance (ANOVA) with Tukey’s post hoc test or Student’s t-test with the level of significance fixed at 0.05.

## Results and Discussion

### NLC characterization

NLC-CLO formulation was obtained as a white-yellowish suspension. The production process reached a yield of 81.5%, which is adequate, considering a laboratory scale.

**Figure 1:**
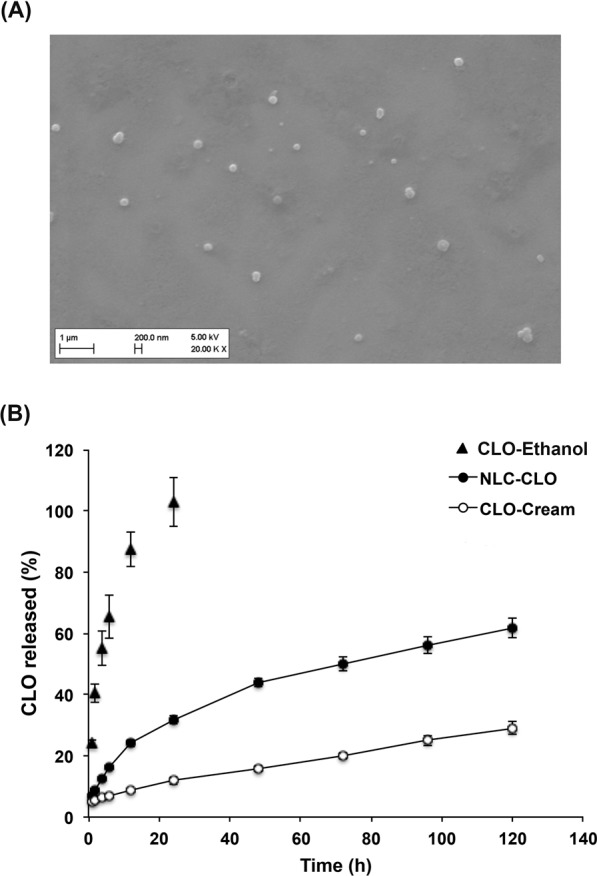
(**A**) Scanning electron microscopy image of nanostructured lipid carriers containing clobetasol propionate; (**B**) Formulations’ release profiles. Results presented as mean ± SD; n = 6. NLC-CLO: nanostructured lipid carrier containing clobetasol propionate; CLO-Ethanol: free drug in EtOH_(aq)_ (45%, v/v); CLO-Cream: commercial cream containing clobetasol propionate.

In a particulate system, an important parameter for transport and release of drugs is the contact surface of the particle. SEM image show NLC-CLO had spherical shape (Fig. 1A), which means the nanoparticles possess an ideal proportion between volume and surface area^27^.

Dynamic light scattering technique revealed NLC-CLO had an average hydrodynamic diameter of 173.80 (±1.35) nm and PdI of 0.158 (±0.032), with monomodal size distribution. Studies with polymeric particles indicated cutaneous application of nanoparticles with sizes between 100 and 200 nm could provide their accumulation into the HF at a depth of 500 to 800 µm^28^. Thus, produced NLC-CLO presented suitable dimension for the proposed application.

NLC-CLO had a zeta potential of −42.20 ± 3.27 mV, which might be advantageous for formulation stability, increasing repulsion and reducing particle agglomeration tendency under storage.

According to DSC results (Supplement 2), CLO melting occurred at high temperatures (T_peak_ = 200.2 °C). In the NLC-CLO, in turn, dehydration of the sample occurred between 30–60 °C and system decomposition were observed after 100 °C, compelled by the degradation of oleic acid and stearic acid which are the most thermosensitive components of the formulation^29^. Moreover, there is no sign of the original CLO crystal in the nanostructure.

Total CLO in the formulation had a concentration of 0.41 ± 0.01 mg/mL. Loss of drug during production is justifiable and predictable, since the microemulsion dilution technique involves multiple steps and laboratory-scale production leads to significant losses during the process due to the small amounts used. EE refers to the amount of drug encapsulated within the particle in relation to the total drug. For NLC-CLO, EE was 80.83 ± 2.28% (p < 0.05). DL, for its turn, reflects the relationship between drug mass and formulation mass in the particle. In our study, under optimal conditions, if 100% of the added drug were incorporated into the nanoparticle, the DL would be 1.54%. For the produced NLC-CLO, DL was 1.24 ± 0.07%, which is quite appropriate, considering the production losses.

Stability data of NLC-CLO are depicted in Table 1. No significant change in hydrodynamic particle diameter was observed in any analyzed sample. However, PdI considerably increased over time for samples stored at 25 °C and 40 °C (p < 0.05), probably due to nanoparticle agglomeration. Zeta potential and pH remained unchanged for all tested conditions. Storage at 40 °C resulted in decreased EE (p < 0.05). In this condition, CLO expulsion from the nanoparticles into the medium may have occurred. At such extreme storage condition (40 °C) a color change from yellow to slightly brownish was observed in all samples. The nanoparticles were therefore stable for at least 30 days when maintained at 5 °C which is sufficient to carry out the proposed study. In subsequent development steps, scale-up studies and drug product stability should be conducted with lyophilized samples.

**Table 1:** Characterization of the formulation of nanostructured lipid carriers containing clobetasol propionate after stability studies.

	Day	Hydrodynamic diameter (nm)	PdI	Zeta potential (mV)	pH	Total CLO (mg/mL)	Total CLO (%)	EE (%)
	0	173.80 ± 1.35	0.158 ± 0.0032	−42.20 ± 3.27	5.56 ± 0.02	0.41 ± 0.01	100 ± 2.47	80.83 ± 2.28
5 °C	7	178.00 ± 4.20	0.164 ± 0.0035	−41.10 ± 3.40	5.55 ± 0.02	0.40 ± 0.01	98.83 ± 2.69	80.98 ± 4.01
	15	176.10 ± 2.50	0.171 ± 0.0120	−39.70 ± 0.90	5.65 ± 0.02	0.41 ± 0.02	100.16 ± 3.79	78.87 ± 4.45
	30	211.40 ± 4.02	0.207 ± 0.0210	−42.10 ± 0.71	5.58 ± 0.02	0.41 ± 0.01	99.13 ± 2.93	82.41 ± 2.61
25 °C	7	189.40 ± 2.62	0.211 ± 0.0230	−40.70 ± 2.08	5.55 ± 0.02	0.41 ± 0.01	99.45 ± 3.40	80.83 ± 2.28
	15	175.50 ± 2.53	0.296 ± 0.0080	−41.00 ± 1.40	5.63 ± 0.02	0.41 ± 0.02	99.90 ± 5.19	78.98 ± 3.45
	30	267.50 ± 2.68	0.497 ± 0.0140	−41.70 ± 3.02	5.50 ± 0.03	0.40 ± 0.01	98.74 ± 3.08	80.62 ± 4.89
40 °C	7	179.10 ± 2.31	0.307 ± 0.0750	−40.70 ± 3.64	5.53 ± 0.02	0.35 ± 0.03	86.05 ± 7.23	77.03 ± 4.48
	15	181.00 ± 2.53	0.222 ± 0.0080	−40.00 ± 1.40	5.50 ± 0.02	0.34 ± 0.02	83.36 ± 3.46	74.31 ± 3.45
	30	171.10 ± 9.89	0.431 ± 0.0620	−38.00 ± 2.65	5.28 ± 0.06	0.34 ± 0.02	83.66 ± 4.74	71.48 ± 5.05

Results presented as mean ± SD; n = 3. CLO: clobetasol propionate; EE: encapsulation efficiency; PdI: polydispersity index.

Samples of NLC-CLO, free-drug in hydroethanolic solution (EtOH 45%, v/v) and commercial cream were submitted to the drug release assay for 120 h. Figure 1B demonstrates free-drug control formulation came into equilibrium in 24 h. The nanoparticles showed sustained release of the drug, reaching a release of about 50% on the third day of experiment. The release profile of the commercial cream was the slowest, indicating the effect of the high viscosity of the commercial formulation compared to the other liquid systems. Also a certain preference of the drug to stay in the cream’s components is very likely to play a role.

### Cell viability

Studies with human HaCat keratinocytes were performed to verify cell viability after exposure to NLC-CLO and free CLO in hydroalcoholic solution. Phosphate buffer pH 7.4 was used as negative control and 2% Triton X-100 as positive control, providing respectively (100.03 ± 7.22 and 0.54 ± 0.35% viability).

Undiluted NLC-CLO samples – containing 0.05% of CLO – were not toxic to cells (113.27 ± 13.97% viability). Indeed, studies have shown NLC are very well tolerated at the cellular level^30,31^ (Supplement 3).

As expected, cellular exposure to the free drug in hydroethanolic solution showed the negative effect of ethanol to the cell culture (20.72 ± 3.33% viability), confirming the necessity of novel formulations for repeated long-term applications.

### NLC follicle targeting effect

Fluorescent NLC were produced for confocal microscopy following *in vitro* and *ex vivo* cutaneous permeation experiments. Therefore, R6G was selected due to its suitable fluorescence properties, in addition to the similarity of the physicochemical properties to CLO, such as molar mass and lipophilicity. Also, NLC-R6G presented similar characteristics to those of the NLC-CLO (size: 188.1 ± 3.5 nm; PdI: 0.146 ± 0.016; zeta potential: −54.4 ± 1.5 mV and formulation pH: 5.5).

**Figure 2:**
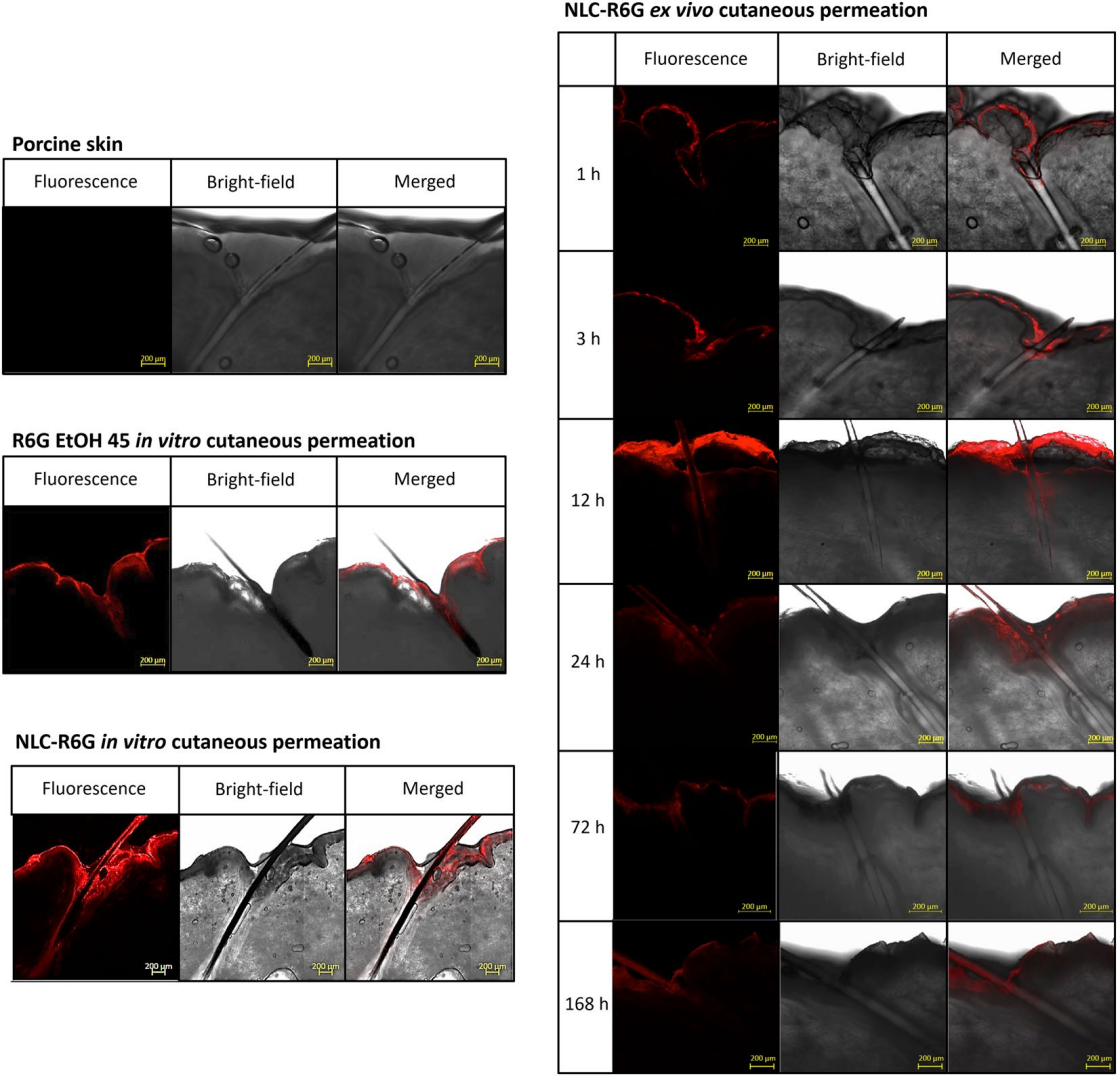
Confocal images of (**A**) hair follicle; (**B**) hair follicle after *in vitro* cutaneous permeation assays in Franz cells with passive application of rhodamine 6 G hydroethanolic solution (R6G EtOH 45) and (**C**) nanostructured lipid carriers containing rhodamine 6G (NLC-R6G). D) Confocal images of hair follicles after *ex vivo* skin permeation assays in Franz cells with passive application of NLC-R6G at 1, 3, 12, 24, 72 and 168 h. Scale bar = 200 μm.

Permeation studies were conducted with the control of 0.05% R6G in 45% (v/v) EtOH_(aq)_ and R6G nanoparticles for 12 h. The dye solution penetrated less into the HF than the nanoparticles, as can be seen in Fig. 2B,C, respectively. NLC-R6G provided fluorescence within the HF, at a depth larger than 900 μm, confirming the specific deposition at the desired target site.

*Ex vivo* skin permeation assays demonstrated the progressive penetration of NLC-R6G through the SC and inside the HF (Fig. 2D).

The observed fluorescence reached the maximum depth into HF after 12 h of assay and could still be observed 7 days after the start of the experiments. Skin samples collected after 12 h indicate that, over time, fluorescence tended to be in more superficial regions. It is possible that over time, the nanoparticles or fluorescent substance, which were deposited more deeply, were metabolized or degraded by the cells of the immune system. Another possible explanation for this phenomenon is the elimination of the substance due to hair growth, which can reach up to about 0.3 mm per day^23^ or elimination of sebum, which *in vivo* can reach 0.1 mg/cm^2^ of skin/h^32^. Such results reveal essential information about the mechanism of drug deposition into the HF from topical preparations, followed by their subsequent elimination.

### *In vitro* cutaneous permeation

#### Passive permeation

**Figure 3:**
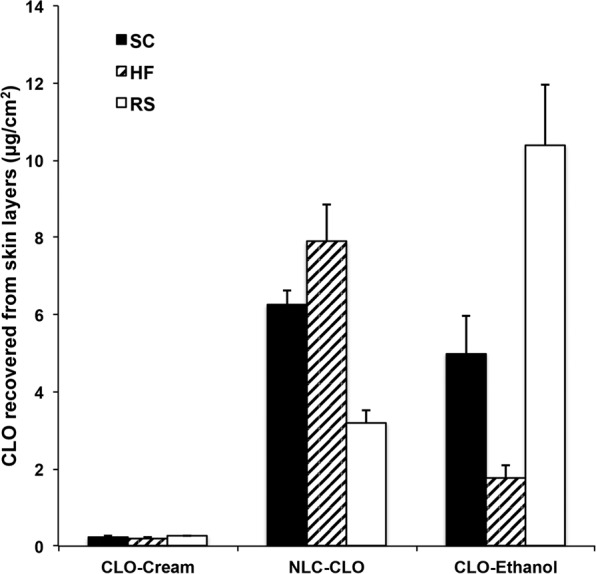
Quantification of clobetasol propionate (CLO) after *in vitro* skin permeation experiments in FD-C with passive application of the formulations. No CLO was observed in the acceptor medium. Results presented as mean ± SD; n = 6. CLO-NLC: nanostructured lipid carrier containing clobetasol propionate; CLO-Ethanol: free drug in EtOH_(aq)_ (45%, v/v); CLO-Cream: commercial cream containing clobetasol propionate; SC: stratum corneum; HF: hair follicles; RS: remaining skin.

In the *in vitro* skin permeation studies, HF accumulation of CLO from the formulations followed the order: commercial cream < hydroethanolic solution < NLC. No CLO was observed in the acceptor solution for any of the analyzed samples (Fig. 3).

NLC-CLO formulation increased drug retention in both SC and RS in comparison to the commercial cream. Studies indicate these types of nanoparticles do not penetrate the epidermis but are primarily retained in the SC or cutaneous appendages^33–35^. NLC lipid content confer a high affinity to the fatty acids naturally present in the SC, from where they release their contents and diffuse into RS^36^. Several studies have shown nanoparticulate formulations promote skin and follicular permeation of their contents more efficiently when compared to conventional formulations^37–39^. Still, NLC-CLO evaluated in the present study demonstrated a remarkable follicular retention: NLC formulation was able to increase the amount of CLO in HF about 4.5- fold compared to the hydroethanolic CLO solution. In relation to the commercial cream, these values reached about 40-fold.

In comparison to the commercial cream, there was higher penetration of CLO from the hydroethanolic solution in all skin layers. This effect was expected because of ethanol’s permeation enhancing effect. Ethanol was employed as a co-solvent, providing higher drug solubility. Such solution was only used as a control, since ethanol would not be the most adequate formulation excipient for inflammatory-related dermatological conditions. In fact, the skin samples exposed to the hydroethanolic solution containing CLO showed a significant change in TEWL values on half of the tested samples (p < 0.0001) (Supplement 4), while no significant variation was observed in skin samples exposed to commercial cream or NLC-CLO. These results confirm the toxicity potential of the hydroethanolic solution, as observed in the cell viability tests. In fact, ethanol possesses dehydration and protein denaturation properties, which can affect the structure of SC and even extract the lipids from this skin layer^40^.

Only 12 h of CLO-EtOH exposition was already sufficient to induce a detectable alteration in the skin barrier function. Thus, it is possible to assume the long-term use of hydroethanolic solution can generate even more severe lesions to the skin, besides the possible sensitization reactions.

Conversely, NLC application allows the formation of a lipid film, occluding the skin and preventing the transepidermal water loss^33^. Such a characteristic might be especially advantageous in cutaneous conditions with SC impairment.

Moreover, results reveal besides higher transdermal permeation, CLO hydroethanolic solution did not provide a HF targeting effect.

Quantified follicular drug retention after NLC-CLO application support what has been observed with the presented confocal microscopy experiments: the chosen nanoparticle formulation is capable of accumulating the studied substance into the HF. Accordingly, even though enhanced follicular accumulation of NLC could be anticipated considering the literature on nanoparticle targeting HF^10,37,41^, such a pronounced difference from the commercial formulation was a surprisingly positive result. *In vitro/in vivo* correlations cannot be directly performed, but dose reduction might be a possibility to attain desired pharmacological effect while avoiding local adverse reactions.

As previously mentioned by Mathes and coworkers^10^, higher follicular uptake was observed when more flexible CLO-loaded nanocapsules were applied to skin, in comparison to two other different polymeric nanocapsules^10^. In that case, approximately 3% of the applied dose was recovered from the HF. In the present study, 500 μL of NLC-CLO formulation containing 0.41 mg/mL of CLO was applied to an exposed skin area of 1.77 cm^2^. Retained drug amounts in the HF was 7.89 ± 0.95 μg/cm^2^, hence, 6.9% of the total applied dose. In addition to a higher affinity for sebum in HF and sebaceous glands, at the skin temperature, NLC may be more flexible than nanocapsules, which may have rendered better targeting potential.

#### Effect of IR irradiation

Several researches have applied IR irradiation to MNP to produce hyperthermia. These particles can absorb light and produce a resonant phenomenon by oscillations, which result in an heating effect^42–44^.

Spectrophotometric scanning demonstrated that at the used concentration of the particles yielded sufficient absorption at the irradiation wavelength used (940 nm). However, the skin’s absorption was high at low wavelength (200–350 nm, Supplement 5). For this reason, in order to selectively induce heating of the formulation and not the skin, Medilas D SkinPulse S laser was used, once it emits IR radiation at 940 nm. A second aspect of this wavelength is that it can penetrate deeper into the tissue and thus might be able to have an effect on and in the hair follicles^45^.

In the present study, hyperthermia was aimed for increasing formulation kinetic energy, consequently nanoparticles Brownian motion. Another possible effect of hyperthermia, hypothesized to increase HF uptake, would be to reduce the stiffness of the sebaceous contents of HF facilitating NLC interaction and penetration.

**Figure 4:**
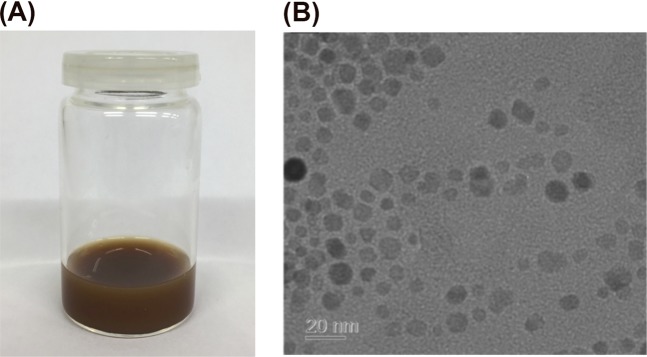
(**A**) Visual impression from the formulation containing magnetite nanoparticles (MNP); (**B**) Transmission electron micrograph of MNP (scale bar = 20 nm).

MNP was obtained as a brown opaque aqueous colloidal dispersion (Fig. 4A). They were stable and could not be separated by a permanent magnet. The concentration of the suspension was 67 mg magnetite per gram suspension. TEM images showed magnetite nanoparticles with a size of approximately 10 nm were produced (Fig. 4B).

Irradiation experiments showed heating from about 1 to 16 °C could be achieved by different combinations of power and time of IR application. When the NLC formulation had MNP added at a concentration of 1 mg/mL, the heating effect was increased by 2.0–3.3-fold (Table 2).

**Table 2:** Achieved heating after laser irradiation (940 nm) at different powers and for different times.

Sample	Power (W)	ΔT (°C)				
		1 min	2 min	3 min	4 min	5 min
NLC-CLO	0.5	0.9 ± 0.1	1.3 ± 0.1	1.6 ± 0.2	2.1 ± 0.3	2.4 ± 0.2
	**1.0**	1.8 ± 0.3	2.7 ± 0.3	**3.3 ± 0.2**	3.8 ± 0.1	4.3 ± 0.2
	1.5	3.8 ± 0.1	5.0 ± 0.2	6.0 ± 0.1	6.9 ± 0.1	7.6 ± 0.2
NLC-CLO + MNP (1 mg/mL)	0.5	2.9 ± 0.4	4.2 ± 0.2	5.2 ± 0.3	6.0 ± 0.4	6.6 ± 0.3
	**1.0**	4.4 ± 0.4	6.1 ± 0.2	**7.5 ± 0.3**	8.3 ± 0.3	9.0 ± 0.4
	1.5	7.9 ± 0.2	10.7 ± 0.5	12.9 ± 0.3	14.3 ± 0.5	15.8 ± 0.2

Results presented as mean ± SD; n = 3. CLO: clobetasol propionate; NLC: nanostructured lipid carries; MNP: magnetite nanoparticles. Selected conditions are highlighted in bold.

In these experiments, extreme heating was not desired as continuous exposure of the skin to high temperatures (>48 °C) has been a factor for hair loss^46^. Thermolysis destroys the HF cells and is, in fact, an approach used in laser photoepilation, aiming to remove unwanted hair^47^. In order to avoid damage to the HF and body thermoregulatory responses (above 40 °C) as infiltration of inflammatory cells in the skin^48^, the heating protocol should be restricted to 40 °C. Considering that skin basal temperature is 32 °C, the use of IR laser at the power of 1.0 W for 3 min was chosen, resulting in final temperatures of about 35.3 ± 0.2 °C for NLC-CLO and 39.5 ± 0.3 °C for NLC-CLO + MNP.

Both NLC-CLO and NLC-CLO + MNP formulations were tested using two different protocols: (i) irradiation at the beginning of the protocol, immediately after application of the formulation and (ii) irradiation at the end of the protocol, after 12 h of the cutaneous *in vitro* permeation.

**Figure 5:**
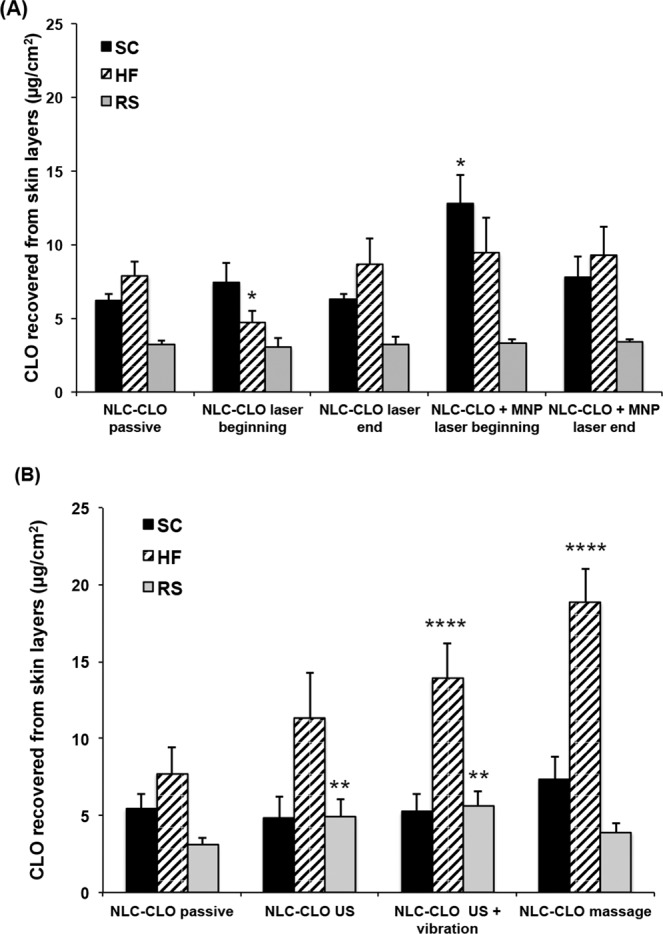
(**A**) Quantification of clobetasol propionate (CLO) after *in vitro* skin permeation experiments in FD-C. Comparison between passive application of NLC and NLC + MNP and infrared laser application immediately after application of each formulation or at the end of 12 h of permeation. No CLO was observed in the acceptor medium. Results presented as mean ± SD; n = 6. *p < 0.05. (**B**) Quantification of clobetasol propionate (CLO) after *in vitro* skin permeation experiments in FD-C. Comparison between passive NLC application, ultrasound (US) with or without vibration and massage. No CLO was observed in the acceptor medium. Results presented as mean ± SD; n = 6. NLC: nanostructured lipid carrier; SC stratum corneum; HF: hair follicle; RS: remaining skin. **p < 0.01; ****p < 0.001.

The hypothesis for the irradiation protocol after 12 h of permeation test was that IR could cause a larger effect after the selective penetration of the nanoparticles into the HF^49^. In none of the trials, however, the laser application protocol at the end of the process resulted in a significant difference over the passive application of the formulation (Fig. 5A).

Nevertheless, the use of the laser at the beginning of the protocol, immediately after application of the formulation, presented statistically significant differences concerning the passive application. For NLC-CLO, the difference was a decrease of 0.6-fold in the accumulation of CLO in the HF, in comparison to passive application. When using NLC-CLO + MNP formulation, in turn, the amount of CLO in the SC was 2.4-fold higher. None of the experiments resulted in CLO in the acceptor medium.

Analysis of particle size distribution and zeta potential of the formulations was performed before and after irradiation without differences being found in particle properties. Therefore, the laser did not modify formulation characteristics, and permeation results should be associated with irradiation effects on the skin, as well as with modifications of the interaction between nanoparticles and skin.

In the experiments using NLC-CLO, the temperature rise was modest (about 3.3 ± 0.2 °C). Studies indicate that moderate heating can stimulate sebaceous glands to expel sebum, which can fill HF, blocking them^33^. Although NLC have an affinity for sebum^40^, they could have been prevented from penetrating more deeply due to physical blockage of the HF in its upper portion.

Instead, in the NLC-CLO + MNP experiments, the higher temperature rise (about 7.5 ± 0.3 °C) may have promoted a much greater fluidization of the sebaceous contents, so that they could have leaked to the SC and deposited on the skin surface^50^. On the surface, the sebum may mix with the SC lipids, forming a film^51^. Thus, NLC may have found a suitable medium for its retention, explaining the higher amount of CLO in such skin layer. Another aspect to be considered is the effect of heating on the SC itself. Temperature elevation, between 35 and 40 °C, provoke a reversible transition in the SC lipids, turning them more fluid^52–54^ and, therefore, more available to mix with topically applied lipophilic substances. As part of the formulation interacts with the SC, less NLC remained available for accumulation into the HF, explaining the lower or equal HF targeting, depending on the temperature, when applying the laser at the beginning of the experiment in comparison to passive permeation.

Nonetheless, even though the use of IR laser has not demonstrated benefit in terms of accumulation of CLO into the HF, the addition of magnetic nanoparticles successfully helped to generate a precise amount of heat to reach HF without causing damage. Additional clinical studies could elucidate whether such controlled hyperthermia would be beneficial to alopecia treatment by other mechanisms than facilitating nanoparticles accumulation into HF, e.g., by increasing blood flow and proliferation of hair matrix trichocytes or by reducing the injurious effects of an inflammatory attack on the HF^55^.

#### Effect of ultrasound and mechanical massage on penetration

Skin samples were analyzed after application of NLC-CLO and high-frequency pulsed US for 3 min (with or without mechanical vibration). Results were compared to a mechanical manual massage also for 3 min.

US application may also elicit hyperthermia^18^. For this, in the present experiment, the US operated in a pulsed manner, which allows the heat dissipation of the medium during the procedure and, therefore, the thermal effect had no contribution in the cutaneous CLO penetration. The temperature was monitored during all tests and did not present any relevant change.

Without mechanical vibration, a significant drug penetration increase was observed in RS (1.6-fold in comparison to the passive application) with no CLO reaching the acceptor medium (Fig. 5B). Although the results pointed to a tendency of increase in follicular retention (1.4 times more CLO retained in comparison to the passive application), there was no statistically significant difference of CLO accumulation in the HF in comparison to the passive application. Conversely, the use of US with vibration resulted in a 1.8-fold increase in CLO retention in HF and 1.8-fold in RS, relative passive application of the formulation, without quantification of CLO in the acceptor medium (Fig. 5B). The accumulation of CLO into the HF, observed after the use of US with mechanical vibration, may be related to the fact that such vibration resembles a semi-automatic massage performed by the device.

Indeed, massaging NLC-CLO formulation on the skin for 3 min resulted in a 2.4-fold increase of CLO in HF, compared to the passive application of the formulation (Fig. 5B). No CLO was observed in the acceptor medium.

Radtke and coworkers^56^ have recently used a stochastic two-dimensional simulation model to demonstrate a ratchet mechanism that explains the penetration enhancement of nanoparticles into HF. The authors found that radially moving hair causes directed nanoparticle transport into the HF, which was most efficient at an optimal driving frequency and optimal particle size^56^. From the three physical stimuli evaluated in the present paper, manual massage was the most effective in targeting HF. The precise fashion in which the hair moves relative to the HF is not completely known for any of the three stimuli applied, but the manual massage may be the one that causes the stronger radial oscillatory hair motion. In this way, hair motion triggered by vibration or a massage may be a more effective way to target the hair follicle than a possible increase in nanoparticle movements as triggered by hyperthermia or cavitation, which most likely had a sub-optimal frequency and, therefore, lead to lower HF deposition.

## Conclusion

Produced NLC formulation demonstrated excellent potential for the accumulation of CLO into the HF, reaching about 40 times more drug at the site of action than the commercial cream used here as a control. The efficacy of the nanosystem for drug targeting to HF was also demonstrated by confocal microscopy images after the application of NLC formulation containing a fluorescent dye. The same formulation was used to demonstrate the penetration and elimination of the dye *ex vivo* for 7 days. The combination of the application of NLC-CLO formulation with the use of physical stimuli demonstrated that among the evaluated methods, IR, US, and massage, the latter seems to be the best approach to specifically increase follicular retention of CLO, more than doubling the amount of the drug into the HF compared to passive NLC-CLO application. IR and US also demonstrated potential results, and more studies are encouraged. Thus, the use of the presented strategies can reduce the dose and frequency of application of the drug and may result in greater safety and efficacy for patients.

## Supplementary information

Supplementary Information

## Data Availability

The datasets generated during and/or analysed during the current study are available from the corresponding author on reasonable request.
