# Supplementary material for: Effect of physical stimuli on hair follicle deposition of clobetasol-loaded Lipid Nanocarriers
Source: Sci Rep. 2020 Jan 13;10:176. doi: 10.1038/s41598-019-56760-w (PMC6957495; doi:10.1038/s41598-019-56760-w)
Supplement: Supplementary file 1 — Supplementary Information [file 41598_2019_56760_MOESM1_ESM.docx]

**Supplement**

**Effect of physical stimuli on hair follicle deposition of clobetasol-loaded Lipid Nanocarriers**

**Tamara Angelo^1,2^, Nesma El-Sayed^2,3^, Marijas Jurisic^2^, Aljoscha Koenneke^2^, Guilherme M. Gelfuso^1^, Marcilio Cunha-Filho^1^, Stephania F. Taveira^4^, Robert Lemor^5^, Marc Schneider^2*^ & Tais Gratieri^1*^**

^1^Laboratory of Food, Drugs, and Cosmetics (LTMAC), University of Brasilia, Brazil

^2^Biopharmaceutics and Pharmaceutical Technology, Saarland University, Saarbrücken, Germany

^3^Department of Pharmaceutics, Faculty of Pharmacy, Alexandria University, Alexandria, Egypt

^4^Laboratory of Nanosystems and Drug Delivery Devices (NanoSYS), School of Pharmacy, Universidade Federal de Goiás (UFG), Brazil

^5^Saarland University of Applied Sciences, Saarbrücken, Germany

^*^Correspondence and requests for materials should be addressed to M.S.

(email: marc.schneider@uni-saarland.de) or T.G. (email: [tgratieri@gmail.com](mailto:tgratieri@gmail.com))

Supplement 1. A) Representative scheme and B) photographs of the differential tape stripping method. 1) Ten adhesive tapes were used to remove the stratum corneum. 2-4) The content of the hair follicle was then removed using cyanoacrylate glue with two adhesive tapes. 5) Finally, the remaining skin was obtained after the edges of the delimited area were cut.

**Supplement 2.** DSC curves of CLO as supplied and NLC-CLO under nitrogen atmosphere.





**Supplement 3.** Cell viability after assay with nanostructured lipid carrier containing clobetasol propionate (NLC-CLO) and free drug in hydroethanolic solution (45%, v/v) (CLO-EtOH 45V). Negative control: phosphate buffer pH 7.4; Positive control: Triton X-100 2%. Results presented as mean ± SD; n = 9.

**

**

**Supplement 4**. Effect of formulations on transepidermal water loss (TEWL) after single skin exposure to formulations for 12 h. A) Clobetasol propionate (CLO) commercial cream; B) nanostructured lipid carrier containing CLO; C) free CLO in EtOH_(aq)_ (45%, v/v). Results presented as mean ± SD; n = 6. Three analyses were performed per sample. Each analysis consisted of one measurement per second, with at least 20 repetitions until the standard deviation was less than or equal to 0.2 in the last five measurements. ^*^p < 0.05; ^***^p < 0.01; ^****^p < 0.0001.

Supplement 5. Representation of absorption spectrum of MNP (magnetite nanoparticles) and skin. MPN diluted 10 times.
